# Toxicology Testing in Fatally Injured Workers: A Review of Five Years of Iowa FACE Cases

**DOI:** 10.3390/ijerph10116154

**Published:** 2013-11-14

**Authors:** Marizen Ramirez, Ronald Bedford, Ryan Sullivan, T. Renee Anthony, John Kraemer, Brett Faine, Corinne Peek-Asa

**Affiliations:** 1 Injury Prevention Research Center, Department of Occupational and Environmental Health, University of Iowa College of Public Health, 105 River St., Iowa City, IA 52242, USA; E-Mails: ronald-bedford@uiowa.edu (R.B.); ryansullivan55@gmail.com (R.S.); corinne-peek-asa@uiowa.edu (C.P.-A.); 2 Department of Occupational and Environmental Health, University of Iowa College of Public Health, 105 River St., Iowa City, IA 52242, USA; E-Mail: renee-anthony@uiowa.edu; 3 Iowa Office of the State Medical Examiner, 2250 S. Ankeny Blvd, Ankeny, IA 50023, USA; E-Mail: John.Kraemer@idph.iowa.gov; 4 Department of Emergency Medicine, University of Iowa Hospitals and Clinics, 200 Hawkins Dr., Iowa City, IA 52242, USA; E-Mail: brett-faine@uiowa.edu

**Keywords:** injury, occupational, fatality, drugs, alcohol

## Abstract

Toxicology testing of fatally injured workers is not routinely conducted. We completed a case-series study of 2005–2009 occupational fatalities captured by Iowa’s Fatality Assessment and Control Evaluation (FACE) Program. The goals of our research were to: (1) measure the proportion of FACE cases that undergo toxicology testing, and describe the factors associated with being tested, and (2) measure the rate of positive toxicology tests, the substances identified and the demographics and occupations of victims who tested positive. Case documents and toxicology laboratory reports were reviewed. There were 427 occupational deaths from 2005 to 2009. Only 69% underwent toxicology testing. Younger workers had greater odds of being tested. Among occupational groups, workers in farming, fishing and forestry had half the odds of being tested compared to other occupational groups. Of the 280 cases with toxicology tests completed, 22% (*n* = 61) were found to have positive toxicology testing. Commonly identified drug classes included cannabinoids and alcohols. Based on the small number of positive tests, older victims (65+ years) tested positive more frequently than younger workers. Management, business, science, arts, service and sales/office workers had proportionately more positive toxicology tests (almost 30%) compared with other workers (18–22%). These results identify an area in need of further research efforts and a potential target for injury prevention strategies.

## 1. Introduction

Traumatic injury claims the lives of over 300,000 workers worldwide, about 4,500 of whom die in the United States each year [1,2]. A wide range of personal and occupational factors are associated with the risk of suffering a fatal workplace injury. Occupational injury studies have focused largely on demographics and worker factors such as job shift or temporary status [3,4]. Among the most prominent yet relatively understudied personal risk factors for occupational injuries is the use of substances such as alcohol and other drugs. 

According to the 2002–2003 National Survey on Drug Use and Health, about 15% of the US working population reported using alcohol or suffering from hang-overs while on the job [5]. Another 8% of US workers report using illicit drugs in the past month. Substance use can lead to reduced physical coordination, poor judgment, and delayed reaction time—all of which impair one’s ability to handle work-related hazards [6]. Relatively few studies have evaluated the association between substance use and traumatic workplace injury, although the role of alcohol and drugs in occupational injury has been discussed in the occupational literature for several decades [4,6].

A number of ethical and methodologic challenges exist to conducting research on this topic. While sophisticated tests are now available to accurately measure levels of substances in human tissue samples, there is no routine testing of alcohol and drug use among workers [7,8]. Prospective research that could implement drug and alcohol screenings to an active working population would encounter significant legal and Human Subjects challenges [9]. In contrast, as part of fatality investigations, traumatic fatal injuries undergo toxicology testing without these same barriers. Hence, the most accurate and accessible measures of alcohol and drugs of workers come from studies of traumatic workplace deaths using secondary surveillance data from coroners or medical examiners. About half a dozen studies using toxicology tests gathered post-mortem are found in the published literature [9,10] with the last US-based studies conducted more than ten years ago [11,12,13,14].

Published studies have all relied on the designation of work-relatedness by the coroner or medical examiner, often using the “injured at work” tick box. This designation is imperfect with both low sensitivity (77%) and positive predictive value (60%) [15,16]. Due to the different definitions used by coroners and medical examiners, young workers and farmers, in particular, are among those frequently misclassified as not work-related [14,16,17]. Among those classified as work-related fatalities, additional misclassification is likely in the designation of occupation because death and law enforcement records focus on documenting usual occupation rather than occupation at time of injury. A final methodologic limitation of these prior studies is selection bias that occurs when evaluating outcomes from screening tests that are not universally administered. Positive bias is likely to occur when those who are suspected to be positive for a drug test, such as males, younger aged groups, transportation-related fatalities are more likely to be screened than others [18]. While prior studies claim that all occupational deaths undergo routine testing, about 15–20% are not tested [11].

To address these limitations and to describe current patterns of substances found in victims of fatal occupational injury, we completed a case-series study of occupational fatalities captured by Iowa’s Fatality Assessment and Control Evaluation Program (IA FACE) [19]. The Iowa FACE program is one of 15 programs funded by the US National Institute of Occupational Safety and Health (NIOSH) to conduct surveillance and investigation of occupational fatalities using team expertise and NIOSH guidelines to identify fatalities. Using IA FACE data on fatally injured workers from 2005–2009, the aims of our research were to: (1) measure the proportion of FACE cases that undergo toxicology testing, and describe the factors associated with being tested, and (2) measure the rate of positive toxicology tests, the substances identified and the demographics and occupations of victims who tested positive.

## 2. Experimental Section

### 2.1. Research Design

This case series study utilized existing data on 427 occupational fatalities collected by the IA FACE program between 1 January 2005 and 31 December 2009. IA FACE relies on multiple sources of data to identify potential cases, including newspaper and other media accounts, the Iowa Division of Labor Services, law enforcement records, and medical examiner reports. Fatality reports are reviewed by a committee of IA FACE investigators and representatives of the Office of the Iowa State Medical Examiner (IOSME) and the Iowa Department of Public Health. 

Iowa FACE investigations collect a number of documents, including: medical examiner reports which have autopsy, pathology, and toxicology reports; police investigation reports; witness and survivor statements; and Department of Transportation reports for transportation-related events. These data are used to determine occupation at time of injury as work-relatedness, as well as to describe circumstances of the event. According to Iowa law, workplace fatalities require a Medical Examiner’s (ME) examination by the state ME or a local ME. Most, if not all ME examinations, include toxicology analysis. A few exceptions are encountered when: (1) the surviving family refuses the examination for personal (e.g., religious) reasons, or (2) the ME did not determine the fatality to be work-related. If, however, the case was a homicide or drowning, caused by a natural disaster, had an undetermined manner of death, or involved unidentified bodies, an autopsy would still be required under state law (Iowa Code 641-127.3(331,691)).

### 2.2. Demographics and Survival Time

Age, gender and race/ethnicity were collected from ME reports or law enforcement records. Races listed in the fatality reports included Asian, Black, Hispanic, Native American and White. Survival time, or time between injury and death, was also collected from ME reports. Since the vast majority of cases died within 24 h of injury, survival time was dichotomized as less than or one day or more.

### 2.3. Toxicology Testing and Positive Status

Cases were considered to have had toxicology testing performed if there was any documentation of results found among the medical examiner reports, autopsy and pathology reports, police and department of transportation reports, and toxicology laboratory reports. Cases were excluded if (1) toxicology testing was performed two or more days after the injury occurred, as the test results would not have been representative of the subject’s condition at the time of the injury, and (2) if substances were administered to victims during life-saving treatments (e.g., lidocaine during resuscitation) which would compromise any toxicology testing. With multiple laboratories and hospitals performing the tests, toxicology results were reported in a variety of formats, including providing detection versus quantification of detected amounts and variability of biological matrix sampled (e.g., tissue, blood, urine). This lack of consistency in reporting levels and small cell sizes found among the few common reporting formats precluded our ability to analyze levels of substances in this study.

Two types of screens were generally utilized: (1) the Drugs of Abuse Panel, which tests for barbiturates, benzodiazepines, cannabinoids, cocaine metabolites, fentanyl, methadone metabolites, opiates, phencyclidine, propoxyphene metabolites, salicylates, and alcohols or (2) the Comprehensive Drug Panel, which includes all substances tested in the Drugs of Abuse Panel as well as analgesics, anesthetics, antibiotics, anticonvulsants, antidepressants, antihistamines, antipsychotics, cardiovascular agents, endocrine agents, gastroenterology agents, narcotics, neurology agents, sedatives/hypnotics, stimulants, and urology agents. Positive tests were determined to be those that detected any screened substance on either of these drug panels. Cases were recoded as negative if positive only for caffeine or cotinine (metabolite of nicotine). For cases that detected both the active ingredient and a metabolite of the same substance (e.g., diazepam and nordiazepam) and cases that detected the same substance in multiple matrices (e.g., alcohol detected in both blood and urine), results were considered to have detected a single positive result for the substance class. Two cases with positive tests for substances known to frequently cross-react with toxicology tests (e.g., pseudoephedrine and amphetamine screening) were considered false positives and were recoded as negative testing results [20,21]. 

Since an aspect of this investigation was to report the prevalence of substances that may have contributed to the fatality, substances were categorized as having the potential to alter the worker’s mental status. Substances in the alcohol, amphetamine, antihistamine, benzodiazepine, cannabinoid, cocaine, opiate, and propoxyphene classes were all considered to have the potential to alter mental status. Antidepressant, cardiovascular, and salicylate classes of substances were identified as not likely to impair mental status. Among the analgesic class, substances such as ibuprofen and acetaminophen were deemed likely to not impair mental status, while other more potent pain relievers (e.g., hydrocodone, tramadol and morphine) were categorized as having the potential to alter mental status.

### 2.4. Occupation, Industry, and Cause of Death Coding

Occupations at the time of injury were assigned Bureau of Labor Statistics (BLS), 2010 Standard Occupational Classification (SOC) [22] codes by three graduate students with discrepancies resolved by a panel of three faculty and staff members in the Department of Occupational and Environmental Health at the University of Iowa. All fatality cases during this study period were categorized into four SOC occupation groups: (1) Construction and Maintenance Occupations; (2) Farming, Fishing and Forestry Occupations; (3) Management, Business, Science and Arts/Service/Sales and Office Occupations; and (4) Production, Transportation, and Material Moving Occupations. 

Business names from the IA FACE files were submitted to the Manta [23] web site to obtain U.S. Census Bureau, North American Industry Classification System (NAICS) industry codes [1]. Industries were further categorized according to the Centers for Disease Control and Prevention’s (CDC) National Occupational Research Agenda (NORA) [24] guidelines. NAICS industry codes were categorized into five groups that correspond with NIOSH’s industry groups identified in their National Occupational Research Agenda (NORA): (1) Agriculture, Forestry and Fishing; (2) Construction/Mining/Oil and Gas Extraction; (3) Manufacturing, Services/Public Safety/Health Care and Social Assistance; (4) Transportation, Warehousing and Utilities; and (5) Wholesale and Retail Trade.

External causes of injury were assigned according to the CDC’s International Classification of Diseases, Tenth Revision (ICD-10) [25]. Causes included were cut/pierce, drowning, falls, fire/flame, firearm, machinery (which includes agricultural machinery), motor vehicle traffic, other pedestrian (not traffic-related), other not traffic-related land transport, other transport (primarily air and water), natural/environmental causes, poisoning, struck by/against (objects or persons), suffocation, and other or unspecified. 

### 2.5. Analyses

Frequencies and percentages were calculated to describe the extent to which toxicology testing was conducted, and among those tested, cases that were positive. First, to examine the factors associated with toxicology testing, we constructed contingency tables and performed chi-square tests between toxicology testing status and sex, age group, race, survival time after injury, occupation, industry, and external cause of death factors. Multivariable logistic regression modeling was performed to identify factors that predicted testing. 

To test for selection bias, we constructed probit Heckman models to assess whether the tested sample was a random sample of the initial larger eligible population for testing [26]. Recall that not all fatality cases underwent toxicology screening, so medical personnel may have selected cases for screening with a bias toward victims who were more likely to be positive. Through a two-stage analysis, the model examines if determinants of having a positive test result are associated with the determinants for being tested in the first place. A very small correlation was measured (−0.04) between the two samples (full sample of victims and tested sample), suggesting no correlation between the samples and, hence, no selection bias for those fatality cases receiving toxicology screens. We, therefore, performed standard logistic regression models with the smaller sample of cases receiving toxicology screens to identify factors associated with positive test outcomes. 

Since occupation and industry were likely to be highly correlated, industry categories were not included in the regression modeling. Due to small cell sizes, for all regression analyses the 15 external cause of death codes were collapsed to three categories: Motor Vehicle Traffic; Other Land Transport (includes animal-drawn vehicles, all-terrain vehicles, railway, and tractors or other agricultural equipment); and All Other causes of death. All statistical analyses were performed using SAS 9.3 statistical software (SAS Institute Inc., Cary, NC) at the 5% level of significance.

## 3. Results and Discussion

### 3.1. Fatal Cases

**Figure 1:**
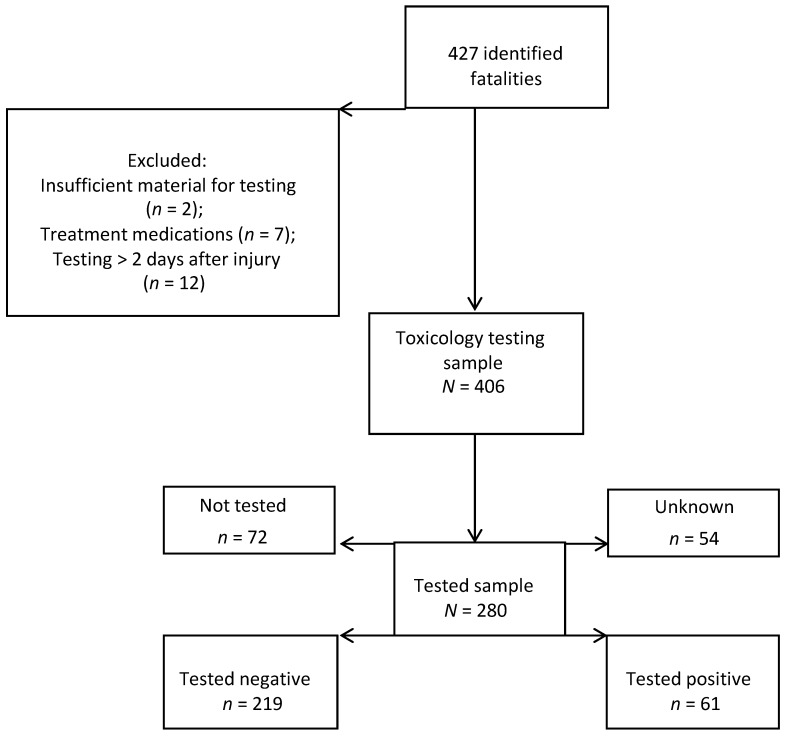
Iowa FACE cases: Flow chart showing inclusions and exclusions.

A total of 427 fatally injured workers were identified from 2005 to 2009. A total of 21 victims were excluded because of insufficient human tissue available for testing (*n* = 2), substances were administered during medical treatment (*n* = 7), or toxicology testing was not completed within two days of the injury (*n* = 12) (Figure 1). 

Of the 406 remaining cases, victims were overwhelmingly male (94.6%) and white (96.4%) (Table 1). Blacks accounted for six (1.7%) of subjects, Hispanics five (1.4%), Asian one (0.3%), and Native American one (0.3%). Victims ranged from seven to 90 years of age, with a median age of 51 years. Nearly 88% (357) of victims died the same day injury occurred. Among the 49 (12.1%) victims who survived one day or more, the longest survival was 366 days. 

**Table 1:** Characteristics of fatally injured workers, Iowa, 2005–2009 (*n* = 406).

	Frequency	Percent
Age (years)		
0–18	6	1.5
19–34	68	16.8
35–49	114	28.1
50–64	142	35.0
65–74	49	12.1
75 and older	27	6.7
Survival since day of injury (days)		
0	357	87.9
> 0	49	12.1
Sex		
Female	22	5.4
Male	384	94.6
Race		
Asian	1	0.3
Black	6	1.7
Hispanic	5	1.4
Native American	1	0.3
White	352	96.4
Missing	41	
Toxicology test performed		
No or Unknown	126	31.0
TestedYes	280	69.0
SOC Occupation Groups		
Construction & Maintenance	85	21.3
Farming, Fishing, & Forestry	135	33.8
Management, Business, Science, & Arts/Service/Sales & Office	57	14.3
Production, Transportation, and Material Moving	122	30.6
Missing	7	
NORA Industry Groups		
Agriculture, Forestry & Fishing	139	35.9
Construction/Mining/Oil & Gas Extraction	61	15.8
Manufacturing	26	6.7
Services/Public Safety/Health Care & Social Assistance	64	16.5
Transportation, Warehousing & Utilities	66	17.1
Wholesale & Retail Trade	31	8.0
Missing	19	
External Cause of Injury		
Cut/Pierce	2	0.5
Drowning	5	1.2
Fall	37	9.1
Fire/Flame	8	2.0
Firearm	14	3.5
Machinery (including agricultural machinery)	18	4.4
MV traffic	98	24.1
Other Pedestrian (not traffic-related)	9	2.2
Other Land Transport (not traffic-related)	93	22.9
Other Transport (primarily air & water)	14	3.5
Natural/ Environmental (primarily weather-related)	11	2.7
Poisoning	7	1.7
Struck By/Against (objects or persons)	33	8.1
Suffocation	18	4.4
Other Specified & Unspecified (not assigned to specific category)	39	9.6

Using SOC codes, a third of victims were farming, forestry and fishing workers (*n* = 135, 33.8%) of whom all but two were farmers followed by production, transportation, and material moving workers (*n* = 122, 30.6%). Using NAICs codes, agriculture, forestry, and fishing was the most frequently identified industry category with 139 (35.9%) cases, followed by transportation, warehousing, and utilities with 66 (17.1%) cases. About 16% of victims worked in services, public safety, health care and social assistance industries (*n* = 64), and mining, and oil and gas extraction industries (*n* = 61). Among the external causes of death, motor vehicle traffic with 98 (24.1%) cases and other land transport with 93 (22.9%) cases each accounted for more than twice as many fatalities as any other external cause of death. Of the 98 motor vehicle deaths, 93 were occupants and four were pedestrians.

### 3.2. Toxicology Testing

Of 406 cases, 280 (69.0%) had toxicology testing performed and 126 (31.0%) had no toxicology reports available (Table 2). Victims less than 34 years old had more than four times the odds (OR = 4.2, 95% CL = 1.6–11.2) and those between 35 and 49 years old had over twice the odds (OR = 2.7, 95% CL = 1.2–6.1) of having toxicology testing performed compared to subjects age 65 and older. Subjects in the 50–64 year old age group were more likely to have had toxicology testing, but the OR (1.6, 95% CL = 0.8–3.2) was not statistically significant. Among the almost 88% of cases who died the same day the injury occurred, the odds of having had some type of toxicology testing was over 13 times greater than for subjects who survived more than one day (OR = 13.3, 95% CL = 5.8–30.8).

SOC occupational groupings were predictive of having had toxicology testing performed. Workers fatally injured in farming, fishing, and forestry occupations had half the odds of having a toxicology test as their counterparts in occupations related to production, transportation, and material moving (OR = 0.4, 95% CL = 0.2–0.8). Although not statistically significant, female subjects were less likely than males to have had toxicology testing performed (OR = 0.5, 95% CL = 0.2–1.6). Compared to work-related fatalities involving motor vehicle traffic, subjects who died as a result of other land transport (OR = 0.5, 95% CL = 0.2–1.3) and all other external causes combined (OR = 0.6, 95% CL = 0.3–1.2) appeared less likely to have had any toxicology testing performed. ORs for each of these independent variables were not statistically significant, however.

**Table 2:** Demographic, occupation, industry, and external cause by toxicology testing status (*n* = 406).

Characteristic	Total N (%)	Toxicology Test performed *n* (row %)	Crude OR (95% CI)	Adjusted OR * (95% CI)
Sex				
Female	22 (5.4)	13 (59.1)	0.6 (0.3–1.5)	0.5 (0.2–1.6)
Male	384 (94.6)	267 (69.5)	1.0 (referent)	1.0 (referent)
Race				
White	352 (96.4)	258 (73.3)	1.0 (referent)	1.0 (referent)
Other	13 (3.6)	10 (76.9)	1.2 (0.3–4.5)	0.6 (0.1–2.7)
Age Group **				
0–34	74(18.2)	60 (81.1)	3.9 (1.9–8.1)	4.2 (1.6–11.2)
35–49	114 (28.1)	84 (73.7)	2.5 (1.4–4.7)	2.7 (1.2–6.1)
50–64	142 (35.0)	96 (67.6)	1.9 (1.1–3.3)	1.6 (0.8–3.2)
65 & older	76 (18.7)	40 (52.6)	1.0 (referent)	1.0 (referent)
Survival since day of injury (days) **				
0	357 (87.9)	270 (75.6)	12.1 (5.8–25.3)	13.3 (5.8–30.8)
>0	49 (12.1)	10 (20.4)	1.0 (referent)	1.0 (referent)
SOC Occupation group **				
Construction & Maintenance	85 (21.3)	63 (74.1)	0.7 (0.3–1.3)	1.2 (0.5–2.9)
Farming, Fishing, & Forestry	135 (33.8)	78 (57.8)	0.3 (0.2–0.6)	0.4 (0.2–0.8)
Management, Business, Science, & Arts/Service/Sales & Office	57 (14.3)	38 (66. 7)	0.5 (0.2–1.0)	0.8 (0.3–2.0)
Production, Transport, Material Moving	122 (30.6)	99 (81.2)	1.0 (referent)	1.0 (referent)
NORA Industry group **				
Agriculture, Forestry & Fishing	139 (35.9)	81 (58.3)	0.5 (0.2–1.2)	
Construction/Mining/Oil & Gas	61 (15.8)	48 (78.7)	1.3 (0.5–3.5)	
Manufacturing	26 (6.7)	20 (76.9)	1.2 (0.3–3.9)	
Services/Public Safety/Health Care & Social Assistance	64 (16.5)	45 (70.3)	0.8 (0.3–2.2)	
Transportation, Warehousing & Utilities	66 (17.1)	52 (78.8)	1.3 (0.5–3.5)	
Wholesale & Retail Trade	31 (8.0)	23 (74.2)	1.0 (referent)	
External Cause of Injury **				
MV Traffic	98 (24.1)	79 (80.6)	1.0 (referent)	1.0 (referent)
Other Land Transport	93 (22.9)	62 (66.7)	0.5 (0.3–0.9)	0.5 (0.2–1.3)
All Other	215 (52.1)	139 (64.7)	0.4 (0.3–0.8)	0.6 (0.3–1.2)

* Logistic regression: Odds ratio adjusted for all other variables in the model; ** *p* < 0.05, calculated using Wald chi-square test.

### 3.2. Positive Toxicology Tests

**Figure 2:**
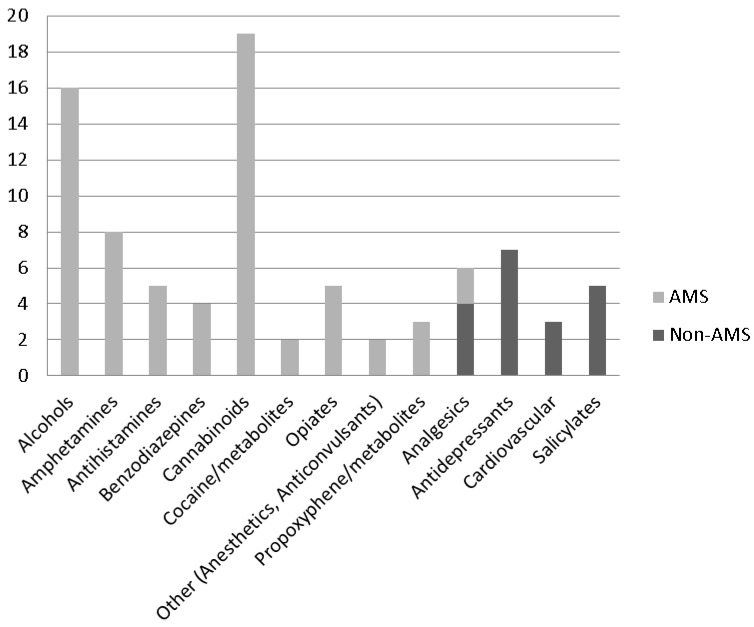
Substance classes detected in toxicology tests of fatally injured workers (*n* = 85).

Among the 280 cases tested, 61 (22%) cases had positive toxicology tests for at least one substance. Over 70% (43) had a single positive result, while 15 cases (24.6%) had two substances detected. Three substances were detected in two (3.3%) cases and in one (1.6%) case, six substances were detected. Exclusive of the stimulants caffeine and cotinine, fourteen substance classes were detected among IA FACE victims (Figure 2). Cannabinoids and alcohols were by far the most frequently detected substance classes, with 19 (22.4%) and 16 (18.8%) detections, respectively. Over 69% (59/85) of the substances detected were considered to have the potential to alter mental status.

AMS = Alter Mental Status; Other = Anesthetics and anticonvulsants classes.

Although few non-white fatality cases had toxicology panels, non-white victims had more positive test results (*n* = 4, 40.0%) compared to white victims (*n* = 55, 21.7%) (Table 3). Although tested at a lower frequency than other age groups, older victims (65+ years) had more frequent positive test results (27.5%) than younger workers (18–22%). Management, business, science, arts, service and sales/office workers had proportionately more positive toxicology tests (almost 30%) compared with other workers (17–22%). Surprisingly, the occupation with the lowest proportion of positive test results was construction and maintenance workers (17%), and 22% of production, transportation and material moving workers had positive drug or alcohol tests. About 30–31% of victims from the manufacturing, services, public safety, health care and social assistance industries were positive for drugs or alcohol. Also striking was the finding that victims who were killed from motor vehicle traffic incidents actually had fewer positive tests (18%) than victims of land transport incidents (26%) or other external causes (22%). Multivariable models comparing the odds of having a positive test were generated, but no estimates were found to be statistically significant either due to small cell sizes or the lack of true associations.

**Table 3:** Demographic, occupation, and industry characteristics by toxicology testing results for IA worker fatalities (*n* = 280).

Characteristic	Positive for any drug		Positive for drug with potential to alter mental status		Total *n* (col %)
	*n* (row %)	*p*-value *	*n* (row %)	*p*-value *	
ALL	61 (21.8%)		50 (17.9%)		280
Sex		0.908		0.616	(100%)
Female	3 (23.1)		3 (23.1)		13 (4.6)
Male	58 (21.7)		47 (17.6)		267 (95.4)
Race		0.175		0.078	
White	55 (21.3)		44 (17.1)		258 (96.3)
Other	4 (40.0)		4 (40.0)		10 (3.7)
Age Group		0.596		0.608	
0–34	13 (21.7)		12 (20.0)		60 (21.4)
35–49	20 (23.8)		17 (20.2)		84 (30.0)
50–64	17 (17.7)		13 (13.5)		96 (34.3)
65 & older	11 (27.5)		8 (20.0)		40 (14.3)
Survival since day of injury (days)		0.168		0.317	
0	57 (21.1)		47 (17.4)		270 (96.4)
>0	4 (40.0)		3 (30.0)		10 (3.6)
SOC Occupation group		0.612		0.500	
Construction & Maintenance	11 (17.5)		9 (14.3)		63 (22.7)
Farming, Fishing, & Forestry	17 (21.8)		14 (18.0)		78 (28.1)
Management, Business, Science, & Arts/Service/Sales & Office	11 (29.0)		10 (26.3)		38 (13. 7)
Production, Transportation, & Material Moving	22 (22.2)		17 (17.2)		99 (35.6)
NORA Industry group		0.531		0.106	
Agriculture, Forestry & Fishing	16 (19.8)		14 (17.3)		81 (30.1)
Construction/Mining/Oil & Gas Extraction	10 (20.8)		10 (20.8)		48 (17.8)
Manufacturing	6 (30.0)		5 (25.0)		20 (7.4)
Services/Public Safety/Health Care & Social Assistance	14 (31.1)		14 (31.1)		45 (16.7)
Transportation, Warehousing & Utilities	11 (21.2)		5 (9.6)		52 (19.3)
Wholesale & Retail Trade	3 (13.0)		2 (8.7)		23 (8.6)
External Cause of Injury		0.505		0.331	
MV Traffic	14 (17.7)		12 (15.2)		79 (28.2)
Other Land Transport	16 (25.8)		15 (24.2)		62 (22.1)
All Other	31 (22.3)		23 (16.6)		139 (49.6)

* *p*-values calculated using Wald chi square test.

### 3.4. Discussion

This study describes the toxicology testing status and results in a series of 427 victims who died on the job in Iowa between 2005 and 2009. Iowa law requires that autopsies be performed in all cases of “work and farm-related deaths unless there is an obvious natural cause of death” (Iowa Code 641-127.3(331,691)). Most, if not all, autopsies of work-related fatalities should include toxicology testing. In our study, however, only a little more than two-thirds of fatally injured workers had toxicology tests performed. Furthermore, certain types of victims were more likely to be tested than others: (1) those who died the same day of their traumatic injury and (2) workers who were less than 50 years of age. Independent of age, agricultural workers were much less likely to undergo toxicological testing than other occupations.

Prior studies report that slightly higher proportions (87–95%) of fatally injured workers underwent toxicology testing [10,11,12,14] compared with our estimate of 69%. This disparity may be due to undercounting of work-related deaths that are then investigated and tested for drugs and alcohol, particularly in studies where only one source of data exists. Most cases identified by coroners and medical examiners underestimated work-related fatalities as defined by federal agencies [15,16]. In rural states like Iowa, misclassification of work-related injuries is particularly problematic for cases of work-related deaths to farmers—a group prone to high misclassification due to the nature of the agricultural work (rural and isolated) and the demographics of farmers (older, often retired or farming as a secondary or part-time job) [14,17]. Similarly, fatally injured workers who die more than 24 h after their injury and older workers may also be more difficult to ascribe the injury as work-related. As an exception, one US national study using the Census of Fatal Occupational Injuries (CFOI) also reported lower rates of testing; toxicology reports were available for only about a fourth of all cases [13]. Our FACE data, like CFOI, is a multiple source surveillance system that likely captures work-related fatalities more reliably than single source studies [19]. Although there are factors associated with the likelihood of being tested (age, occupation, survival time), we found no evidence of selection bias. Hence, while younger workers were more likely to be tested than older workers and farmers less likely to be tested than other occupations, fatal injury cases with toxicology testing were neither more nor less likely to be positive than the untested cases. Our findings still underscore the need for complete testing of all occupational fatalities, nonetheless. In fact, our reduced sample size of tested victims (from *n* = 406 to *n* = 280) led to power limitations in our analyses and thus an inability to construct multivariable models to identify predictors for positive test status. Furthermore, sample collection sites (femoral, heart, subclavian, vitreous, antemortem, *etc.*) were not controlled, so the ability to assess postmortem redistribution was not able to be taken into account. Postmortem redistribution could be a factor in determining a positive or negative result if the concentration for a particular drug is near the reporting limit.

A number of findings related to the toxicology test results are noteworthy. Of those tested, 22% were positive for alcohol or other substances, an estimate just slightly higher than those reported (17%–20%) in prior research conducted in the U.S. and Australia [10,11,12,13,14]. Alcohol and cannabinoids were among the most frequently detected substances, which is also rather consistent with prior studies which have consistently identified alcohol as the top substance found in toxicology reports of fatally injured workers [9,10,11,12].

Unlike prior studies construction and maintenance workers in our study had the lowest proportion of positive test results compared with other occupations [13]. Furthermore, in all other studies previous to ours, deaths attributed to motor vehicle crashes were most frequently positive for alcohol and/or drugs compared to other external causes [10,11,12,13,14]. In our study, positive toxicology tests were more often found among deaths due to other land transport or other causes. A number of explanations are possible. First, our results are limited to the population of Iowa workers, who, in fact, may differ from workers in other settings. Second, the choice of drugs for testing was not consistent across studies. The Australian study, for example, limited its drug screenings to alcohol, cannabinoids, and methamphetamines [10]. The Texas study included drugs used for medical treatment [12]. Finally, studies used different tissue samples for testing. In our study, resulting positive toxicological testing from any testing matrix, including blood, vitreous samples and/or urine, was acceptable. Our goal was to identify the presence of any substance in all types of human samples used in toxicology testing of fatally injured workers.

A result of interest was that workers 65 years or older tested positive for substances more frequently than younger workers, although differences were not statistically significant. With the greying of baby boomers comes a growing number of older workers, who comprised about 19% of the U.S. workforce in 2009. Older workers suffer the highest rate of fatal injury compared with their younger counterparts, and are at high risk of fatal injury [27,28]. Although we cannot determine if the drugs indeed led to fatal injury, use of drugs and prescription drugs for this age group, in particular, may impair judgment and motor coordination. Hence, further research particularly with a larger sample size is needed to determine if older workers have indeed a high incidence of drug-related fatalities.

The study has a few limitations. Classification of external causes was quite broad due to our small sample of tested and positive cases. Positive toxicology test results do not necessarily mean that workers were impaired at the time of the traumatic injury [12]. In fact, residual metabolites or traces of substances remain in the body for weeks after ingestion. Toxicology tests were also conducted in at least 13 laboratories across IA and in four neighboring states which may lead to some level of misclassification due to different samples collected for testing or different reference levels used to ascertain positive test status. Finally, while we capture prevalence of drugs and alcohol in fatally injured workers, we cannot generalize our estimates to non-occupational fatalities or even populations currently active in the workforce.

## 4. Conclusions

Despite challenges in classification and capturing work-related fatalities, surveillance of occupational fatalities is critical for prevention. Data collected via surveillance efforts like toxicology tests serve as an important source of information for identifying trends on drug and alcohol use among fatally injured victims. In this study, we found that about one in five workers killed on the job tested positive for alcohol or other drugs. Substance abuse interventions for workers, particularly in rural states like Iowa, should focus on alcohol and cannabinoid use across multiple occupational groups and industries and potentially among older workers.
